# Modulation of Tamoxifen Cytotoxicity by Caffeic Acid Phenethyl Ester in MCF-7 Breast Cancer Cells

**DOI:** 10.1155/2016/3017108

**Published:** 2015-11-30

**Authors:** Tarek K. Motawi, Samy A. Abdelazim, Hebatallah A. Darwish, Eman M. Elbaz, Samia A. Shouman

**Affiliations:** ^1^Department of Biochemistry, Faculty of Pharmacy, Cairo University, Cairo 11562, Egypt; ^2^Department of Cancer Biology, National Cancer Institute, Cairo University, Cairo 11796, Egypt

## Abstract

Although Tamoxifen (TAM) is one of the most widely used drugs in managing breast cancer, many women still relapse after long-term therapy. Caffeic acid phenethyl ester (CAPE) is a polyphenolic compound present in many medicinal plants and in propolis. The present study examined the effect of CAPE on TAM cytotoxicity in MCF-7 cells. MCF-7 cells were treated with different concentrations of TAM and/or CAPE for 48 h. This novel combination exerted synergistic cytotoxic effects against MCF-7 cells via induction of apoptotic machinery with activation of caspases and DNA fragmentation, along with downregulation of Bcl-2 and Beclin 1 expression levels. However, the mammalian microtubule-associated protein light chain LC 3-II level was unchanged. Vascular endothelial growth factor level was also decreased, whereas levels of glutathione and nitric oxide were increased. In conclusion, CAPE augmented TAM cytotoxicity via multiple mechanisms, providing a novel therapeutic approach for breast cancer treatment that can overcome resistance and lower toxicity. This effect provides a rationale for further investigation of this combination.

## 1. Introduction

Breast cancer is the most prevalent cancer among the female population [1]. Despite the evolution in treating breast cancer, it is still the principal cause of cancer death in females [1, 2].

Tamoxifen (TAM) is a widely used antiestrogenic drug for treating breast cancer patients [3]. Although there are many satisfying outcomes from the endocrine therapy with TAM, not all treated patients get the hopeful result. High concentration of TAM showed several hormonal dependent and independent effects. TAM can induce apoptosis of cancer cells via the involvement of a mitochondria-dependent pathway, the amendment of signaling proteins such as protein kinase C, and/or the upregulation of p53 [4]. Hence, a combination of lower concentrations of TAM with other drugs of synergistic antitumor effect might be of priority in the therapy of breast carcinomas.

Caffeic acid phenethyl ester (CAPE) is a polyphenolic compound existing in numerous medicinal plants and in propolis [5]. It is subjected to the action of blood esterase due to its aryl ester structure. The pharmacokinetic profiles of CAPE in rat tissues showed high values of volume of distribution and short elimination half-life after its systemic administration [6].

CAPE has a diversity of important biological activities including antibacterial, antiviral, and anticancer ones [7]. Moreover, at low doses, CAPE inhibits lipid peroxidation [8] and shows antioxidant activities [9].

Several reports have shed light on the impact of CAPE on cell cycle progression, cell proliferation, induction of cell cycle arrest, and apoptosis [10]. The* in vitro* and* in vivo* inhibitory effects of CAPE were predictable in plenty of cancer models, such as colon [11], lung cancers [12], and pancreatic carcinoma [13]. A remarkable finding is the ability of CAPE to exhibit differential toxicity against tumor cells without affecting normal cells. In this context, CAPE has no cytotoxic effect on normal nonmalignant cells as MCF-10A mammary cells [5, 14].

The competency to increase response and reduce chemoresistance of cancer therapeutics via the use of the combination therapy might thus be a significant advantage for cancer patients. Combination therapies promoting the effectiveness of TAM have been previously investigated in several studies, using compounds as vitamin E [15] and green tea [16].

Accordingly, this study examined the efficacy of TAM and CAPE with regard to multiple targets as apoptosis, autophagy, angiogenesis, and oxidative stress in breast cancer cells.

## 2. Materials and Methods

### 2.1. Drugs

TAM was a kind gift from El Amirya Pharmaceuticals Company (Alexandria, Egypt). It was dissolved in dimethyl sulfoxide (DMSO) at concentration 1 : 1 and stored at −20°C.

CAPE was purchased from Sigma-Aldrich Chemical Co. (ST. Louis, MO, USA). The compound was dissolved in DMSO at 100 mM concentration and stored at −20°C. Serial dilutions of both drugs were made in cell culture medium just prior to use, so that the final concentration of DMSO was about 0.1% (v/v).

### 2.2. Chemicals

Fetal bovine serum (FBS, F6178), L-glutamine, penicillin/streptomycin antibiotic, RPMI-1640 medium (R8758), and trypsin-EDTA were purchased from Biowest, France. Agarose and DMSO were purchased from Sigma-Aldrich Chemical Co., USA. Antibodies used for the detection of caspase-9 (primary mouse anti-human caspase-9 monoclonal antibody), microtubule-associated protein light chain 3- (LC3-) II (primary rabbit LC3-II oligoclonal antibody), and *β*-actin (primary rabbit anti-human *β*-actin monoclonal antibody) were obtained from eBioscience (Austria), Invitrogen (USA), and Sigma-Aldrich (USA), respectively. The primer sequences for Bcl-2, Beclin 1, vascular endothelial growth factor (VEGF), and GADPH were supplied by R&D systems (Minneapolis, MN, USA). Thermo Scientific Gene JET RNA Purification Kit (UK), Thermo Scientific RevertAid First Strand cDNA Synthesis Kit (UK), and Thermo Scientific Maxima SYBR Green/ROX qPCR Master Mix kit (UK) were used in quantitative real time PCR analysis. Caspase-3 activity was measured using the colorimetric assay kit (R&D systems, USA). DNA fragmentation was performed by QIAamp DNA Mini Kit (QIAGEN, USA) using a suitable DNA marker (Gibco, BRL, Life technologies, USA). Other reagents were of analytical grade or the highest quality available.

### 2.3. Human Cancer Cell Line and Cell Culture

Human breast cancer cell line MCF-7 was obtained from the American Type Culture Collection (ATCC, Manassas, VA). MCF-7 cells were cultured in RPMI-1640 medium supplemented with heat-inactivated 10% FBS, 100 U/mL penicillin, 100 *μ*g/mL streptomycin, and 2 mM L-glutamine. Cells were incubated at 37°C in a humidified 5% CO_2_ incubator.

### 2.4. Cytotoxicity Assay

Cytotoxicity was determined using SRB method as described by Skehan et al. [17]. In brief, cells were seeded at a density of 3 × 10^3^ cells/well in 96-well microtiter plates. They were left to attach for 24 h before incubation with drugs. The old medium was discarded and was replaced with a fresh one containing drugs added alone or simultaneously with the specified concentrations.

In brief, the cells were treated with different concentrations of TAM (10, 20, 30, 40, and 50 *μ*M), CAPE (0.1, 1, 10, 100, and 200 *μ*M), or their combination. Two different regimens have been designed to approach the most effective concentrations:

- Cross matching combination regimen, TAM (10, 20, 30, 40, and 50 *μ*M) with matched reversed doses of CAPE (0.1, 1, 10, 100, and 200 *μ*M).
- Fixed dose combination regimen, 10 *μ*M of TAM with different concentrations (0.1–200 *μ*M) of CAPE.

For each sample, three wells were used for every concentration and incubation was continued for 48 h. The same volume (200 *μ*L/well) of DMSO (1% v/v) was used as the vehicle control. At the end of incubation, cells were fixed with 20% trichloroacetic acid (TCA), stained with 0.4% sulforhodamine-B (SRB), and rinsed with 1% acetic acid. The bound protein stain was solubilized with Tris base (10 mM, pH 10.5) and the optical density (OD) of each well was measured spectrophotometrically at 570 nm using ELISA microplate reader (TECAN sunrise, Germany). The experiment was repeated 3 times and the mean values were estimated as fraction of cell survival as follows: OD (treated cells)/OD (control cells).

The IC_50_ value (the required concentration to produce 50% inhibition of cell growth) of each drug was calculated using sigmoidal dose response curve-fitting models (GraphPad Prism software, version 5).

### 2.5. Evaluation of Drugs Interaction

The interaction between CAPE and TAM was evaluated by the isobologram equation: the combination index (CI) = *d*1/*D*1 + *d*2/*D*2 [18].

*d*1 and *d*2 signify the respective concentrations of TAM and CAPE used in combination to produce a fixed level of inhibition, while *D*1 and *D*2 represent their concentrations that are alone able to produce the same magnitude of effect. If “CI” is less than 1, the effect of combination is synergistic, whereas if CI = 1 or >1, the effect is additive or antagonistic, respectively.

### 2.6. Determination of TAM Uptake by MCF7 Cells

MCF7 cells 10 × 10^3^/well were seeded in RPMI-1640 medium and left for 24 h. The plate was divided into 2 groups as follows:

- Group I was treated with 10 *μ*M TAM.
- Group II was treated with 10 *μ*M TAM and 4 *μ*M CAPE.

The medium was then aspirated after 0, 2, 4, and 24 h intervals and centrifuged and the supernatant was stored at −20°C till HPLC assay.

### 2.7. Sample Extraction and Preparation for Liquid Chromatography-Tandem Mass Spectrometry

200 *μ*L of the medium was mixed thoroughly with 200 *μ*L acetonitrile (Alliance Bio, USA) and centrifuged at 1400 rpm for 15 min at 4°C. 10 *μ*L of the resultant clear supernatant was then injected into AB SCIEX LC/MS/MS system (AB SCIEX 3200 Q TRAP, Germany) equipped with electrospray ionization (ESI) source and an Agilent 1260 affinity HPLC system, consisting of a vacuum degasser, a binary pump, and an autosampler to determine the concentration of TAM. Analyst 1.5.2 software was used for data acquisition and processing. The analytical column used was Agilent Poroshell 120-C18 (50 mm × 3 mm × 2.7 *μ*m, Agilent, Germany) at 25°C. The mobile phase consists of 0.1% formic acid/water (solvent A) and 0.1% formic acid/acetonitrile (solvent B), delivered at a flow rate of 0.5 mL/min. Mass spectrometric analysis was performed in the positive ion mode.

### 2.8. Western Blotting Analysis

Cells were seeded, cultured, and treated with TAM and CAPE and their combination for 24 h and 48 h. At time of harvest, control and treated cells were collected and lysed in the lysis buffer (150 mM NaCl, 10 mM Tris, 0.2% Triton X-100, 0.3% NP-40 (nonyl phenoxypolyethoxyl ethanol), 0.2% Na_3_VO_4_, and protease inhibitor cocktail, pH 7.4) (Bio Basic Inc., Canada). After centrifugation at 14,000 rpm for 15 min (at 4°C), the protein concentration in the supernatant was measured by Bradford method using Coomassie Protein Assay Kit (Pierce, USA) [19]. Proteins were then separated using 10% sodium dodecyl sulphate polyacrylamide gel electrophoresis (SDS-PAGE). Protein was transferred onto a polyvinylidene difluoride (PVDF) membrane, which was blocked with 5% w/v nonfat dry milk and incubated overnight with the specific primary antibodies {mouse anti-human for caspase-9 monoclonal antibody (1 : 1000), rabbit LC_3_-II oligoclonal antibody (1 : 500), and rabbit anti-human *β*-actin monoclonal antibody (1 : 1000)}. This step was followed by incubation with the appropriate diluted secondary antibody (1 : 2000 in PBS-T) for 1 h at room temperature. The blots were developed with Amersham ECL western blotting detection reagents and analysis system (GE Healthcare, UK) according to the manufacturer's protocol. Protein loading was corrected for *β*-actin and quantitation of band intensity was performed using Image J Software.

### 2.9. Determination of the Enzymatic Activity of Caspase-3 in Cell Lysate

Caspase-3 activity was measured colorimetrically using Caspase-3 Colorimetric Assay kit (R&D, USA, Catalog number BF3100) according to the manufacturer's instructions based on the method of Fernandes-Alnemri et al. [20]. The cleavage of the peptide by the caspase released the chromophore pNA, which can be quantitated spectrophotometrically at a wavelength of 405 nm. Caspase-3 activity was expressed as optical density.

### 2.10. Flow Cytometric Analysis of FITC Annexin V Staining

FITC Annexin V Apoptosis Detection kit (BD Bioscience Pharmingen, San Jose, CA) was used as per manufacturer's recommendation. Approximately 5 × 10^5^ cells were plated in T-75 flasks on Day −1 and left to adhere. On Day 0 the medium was replaced with fresh standard medium, and 10 *μ*M TAM, 4 *μ*M CAPE, or both of them were added. Untreated control received ethanol solvent (0.1%). Cells were harvested after 48 h (Day 2). Cells were trypsinized and then washed in PBS and centrifuged at 1200 rpm for 5 min. The pellets were resuspended in 100 *μ*L of staining solution (containing annexin V-fluorescein and propidium iodide in buffer) and were mixed gently and incubated for 15 min at room temperature (15–25°C) in the dark. Finally, 400 *μ*L of binding buffer was added. FACScan analysis was performed using a Becton Dickinson FAC Scan analyzer (Becton Dickinson, Heidelberg, Germany).

### 2.11. DNA Fragmentation Analysis

DNA was extracted using QIAamp DNA Mini Kit according to the manufacturer's instructions. DNA was electrophoresed and visualized under ultraviolet light using 0.8% agarose gel stained with ethidium bromide.

### 2.12. Quantitative Real Time PCR Analysis

Total RNA was extracted from cell culture by utilizing Thermo Scientific Gene JET RNA Purification Kit, following the manufacturer's protocol. cDNA was generated with M-MuLV reverse transcriptase using Thermo Scientific RevertAid First Strand cDNA Synthesis Kit. For real time PCR quantification, Thermo Scientific Maxima SYBR Green/ROX qPCR Master Mix kit was used. Briefly, in a 25 *μ*L reaction volume, 12.5 *μ*L of master mix, 2.5 *μ*L of primer assay, and 10 *μ*L of template cDNA (100 ng) were added to each well. Sequences of primers were described in Table 1. The PCR plate was subjected to 40 cycles of the following conditions: PCR activation at 95°C for 5 min, denaturation at 95°C for 5 sec, and annealing/extension at 60°C for 10 sec.

The values of RT-PCR products were normalized with respect to GAPDH and then compared to controls. The relative expression was calculated from the 2^−ΔΔCT^ formula [21].

### 2.13. Determination of Glutathione (GSH)

Reduced glutathione was determined as described by Ellman [22]. In brief, cells were collected by trypsinization after treatment with TAM, CAPE, and their combination, as well as the control. Samples were then centrifuged at 1200 rpm for 5 min and the resultant cell pellet was suspended in 1 mL saline. Next, 500 *μ*L of cell suspension was mixed well with 25 *μ*L trichloroacetic acid (TCA) and the tubes were centrifuged at 3000 rpm for 10 min at 4°C. 100 *μ*L of the resultant supernatant was mixed thoroughly with 850 *μ*L of phosphate buffer followed by addition of 50 *μ*L Ellman's reagent. After 5 min, the absorbance was measured spectrophotometrically at 405 nm against a blank. Glutathione content was expressed as nmoles of GSH/mg protein.

### 2.14. Determination of Nitric Oxide (NO)

Nitric oxide produced in cell culture media was estimated spectrophotometrically [23]. Briefly, cells were collected by trypsinization after treatment with TAM, CAPE, and their combination as well as the control. Next, 50 *μ*L of zinc sulfate solution was added to 250 *μ*L media and centrifuged at 17000 rpm. The resultant supernatant was treated with vanadium chloride (0.8% in 1 M HCl) and Griess reagent (prepared by mixing equal volumes of N-1-(naphthyl)ethylenediamine {0.1% in bidistilled water} and sulfanilamide {2% in 5% HCl}). Total NO content was expressed as *μ*g/mL.

### 2.15. Statistical Analysis

Data were expressed as means ± SD. Differences among groups were tested using one-way analysis of variance (ANOVA) followed by a Tukey post hoc correction for multiple comparisons using SPSS (version 17.0). Significant differences were considered at *P* value < 0.05. All figures were established using GraphPad Prism, version 5.

### 2.16. Ethical Aspects

There is no need for ethical approval or informed consent since all experiments were carried out using MCF-7 breast cancer cell line.

## 3. Results

### 3.1. Growth Inhibition of TAM, CAPE, and Their Combination against MCF-7 Cell Line

**Figure 1:**
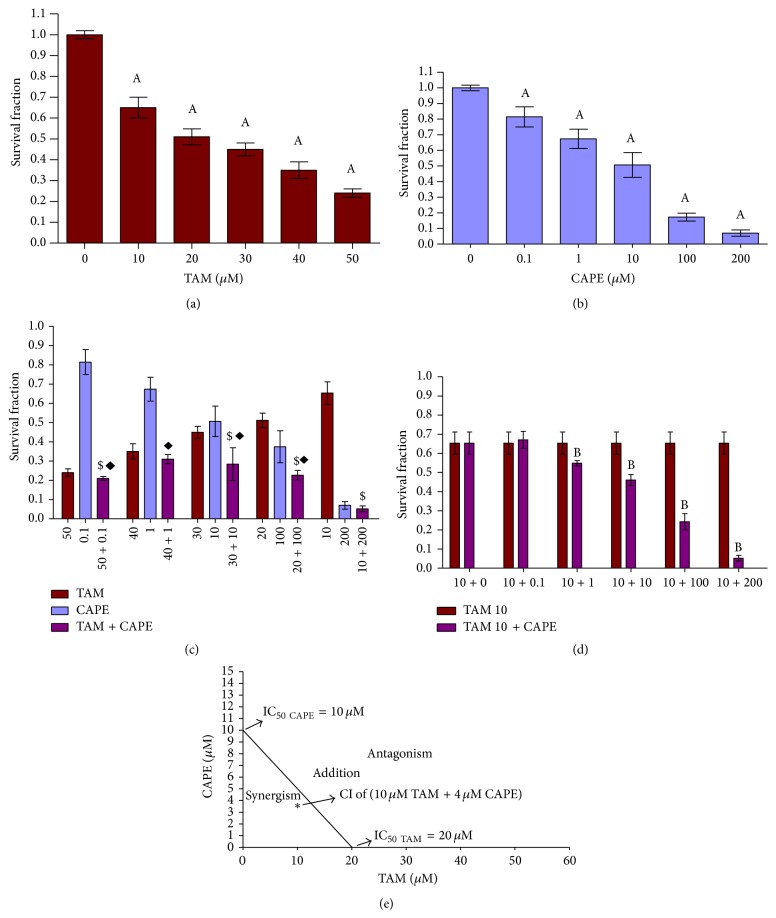
Survival fraction in MCF-7 cells after 48 h treatment with TAM, CAPE, and their combination. Cells were treated with various concentrations of (a) TAM, (b) CAPE, (c) TAM + CAPE, and (d) 10 *μ*M TAM + CAPE for 48 h. (e) Isobologram analysis of MCF-7 cell growth inhibition by TAM and CAPE after 48 h of treatment. The IC_50_ value of each drug after 48 h is plotted on the axes; the solid line represents the additive effect, while the asterisk (*∗*) located below the connecting line points to the synergistic effect of the concentrations of TAM and CAPE used in the combination. Results were expressed as means ± SD of 3 independent experiments performed in triplets. *P* value < 0.05 is considered significant. A: significantly different from the respective concentration of control. B or $: significantly different from the respective concentration of TAM. ◆: significantly different from the respective concentration of CAPE.

Figure 1(a) shows the effect of different concentrations of TAM on the survival fraction of MCF-7 cells after 48 h exposure. As evident, there was a significant dose dependent decrease in the survival fraction compared to each respective control value. At 50 *μ*M, maximum cytotoxicity (76%) of TAM against MCF-7 was reached, whereas the IC_50_ value was obtained at 20 *μ*M.

Figure 1(b) depicts the effect of treating MCF-7 cells with different concentrations of CAPE. After 48 h, the number of surviving cells was significantly decreased in a dose dependent manner compared to the respective control values. The effect of CAPE on cell survival reached its maximum (93%) at 100 *μ*M, with IC_50_ value of 10 *μ*M.

When examining the cytotoxic effect of different combinations of TAM and CAPE (Figure 1(c)), we observed some cytotoxic actions upon decreasing the concentration of TAM and increasing that of CAPE. When combining the smallest dose of TAM (10 *μ*M) with different concentrations of CAPE, the IC_50_ of CAPE decreased to a value ranging between 1 *μ*M and 10 *μ*M (Figure 1(d)). An isobologram analysis illustrated the synergistic effect of 10 *μ*M TAM and 4 *μ*M CAPE (CI = 0.9).

### 3.2. Effect of CAPE on the Cellular Uptake of TAM

**Figure 2:**
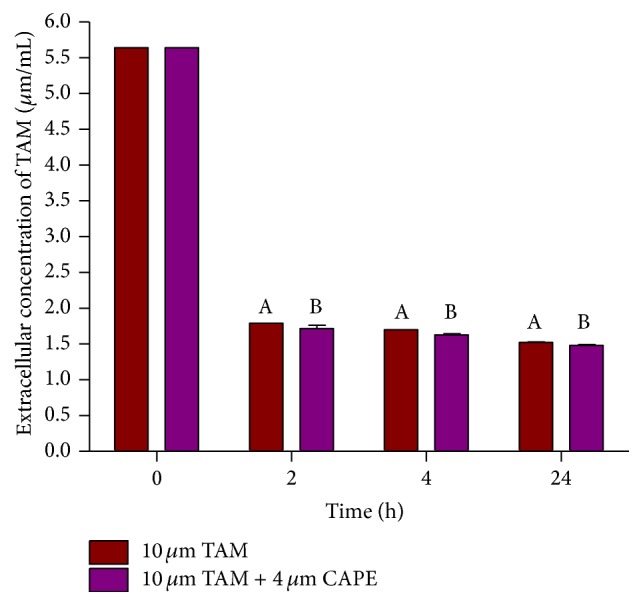
Effect of CAPE on cellular uptake of TAM from the culture medium after different time intervals. Results were expressed as means ± SD of 2 independent experiments. *P* value < 0.05 is considered significant. A: significantly different from TAM alone at *P* < 0.05. B: significantly different from TAM + CAPE at *P* < 0.05.

Figure 2 showed that majority of the TAM was taken by the cells after 2 h treatment. Meanwhile, no significant change in cellular uptake of TAM was achieved upon cotreatment with CAPE, as compared with the respective TAM treated group in all the studied time intervals.

### 3.3. Effect of TAM, CAPE, and Their Combination on Caspase-9 and LC3-II Protein Levels in MCF-7 Cell Line

**Figure 3:**
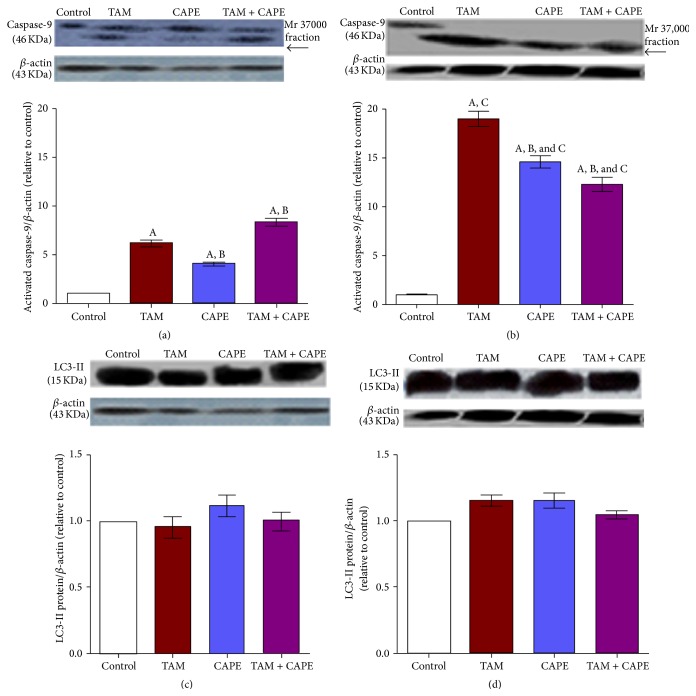
Western blot of caspase-9 and LC3-II in MCF-7 cells. Western blot analysis of caspase-9 and LC3-II after 24 h (a and c) and 48 h (b and d) treatments with TAM (10 *μ*M), CAPE (4 *μ*M), and their combination. Results were expressed as means ± SD of 3 independent experiments performed in triplets. *P* value < 0.05 is considered significant. A: significantly different from the respective concentration of control at *P* < 0.05. B: significantly different from the respective concentration of TAM at *P* < 0.05. C: significant difference of 48 h treatment from the respective concentration at 24 h at *P* < 0.05.

As illustrated in Figures 3(a) and 3(b), incubation of MCF-7 cells with 10 *μ*M TAM, 4 *μ*M CAPE, and their combination for different time intervals (24 h and 48 h) caused activation and subsequent cleavage of caspase-9. On the other hand, no change in the protein level of LC3-II was detected.

### 3.4. Effect of TAM, CAPE, and Their Combination on Caspase-3 Activity in MCF-7 Cell Line

**Figure 4:**
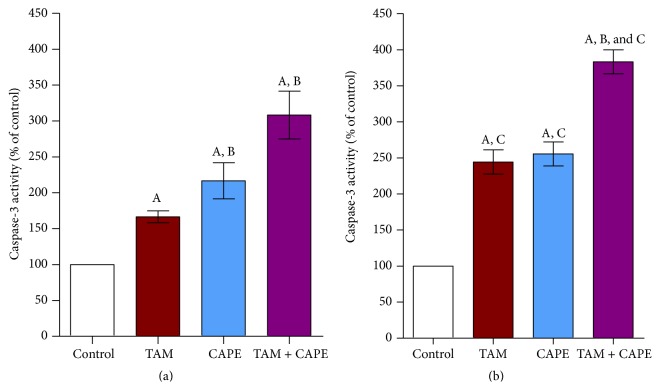
Caspase-3 activity in MCF-7 cells. Caspase-3 activity after 24 h (a) and 48 h (b) treatments with (10 *μ*M), CAPE (4 *μ*M), and their combination. Results were expressed as means ± SD of 3 independent experiments performed in triplets. *P* value < 0.05 is considered significant. A: significantly different from the respective concentration of control at *P* < 0.05. B: significantly different from the respective concentration of TAM at *P* < 0.05. C: significant difference of 48 h treatment from the respective concentration at 24 h at *P* < 0.05.

Treatment of MCF-7 cells with 10 *μ*M TAM produced 1.5- and 2.5-fold increases in caspase-3 enzymatic activity after 24 h and 48 h, respectively, as compared to the control values. Furthermore, 4 *μ*M CAPE exhibited approximately 2.5-fold increases at both treatment periods compared to the control groups. Upon treating the cells with both TAM and CAPE, the activity of caspase-3 was considerably enhanced almost 3- and 4-fold, respectively, as compared to the control values (Figures 4(a) and 4(b)).

### 3.5. Effect of TAM, CAPE, and Their Combination on Apoptosis as Detected by Annexin Binding Assay

**Figure 5:**
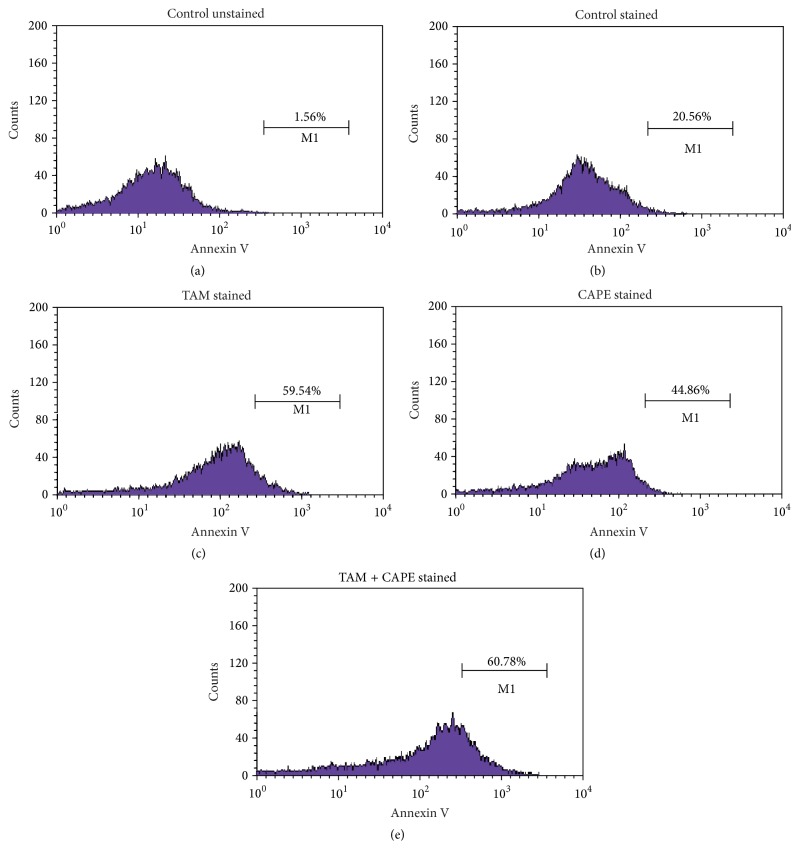
Flow cytometric analysis of TAM, CAPE, and their combination in MCF-7 after 48 h treatment. (a) Untreated unstained MCF-7 cells. (b) Untreated stained MCF-7 cells. (c) MCF-7 cells treated with 10 *μ*M TAM for 48 h. (d) MCF-7 cells treated with 4 *μ*M CAPE for 48 h. (e) MCF7 cells treated with both drugs for 48 h.

As shown in Figure 5, TAM, CAPE, and their combination enhanced apoptosis of MCF7 cancer cells by 59.54%, 45%, and 61%. It is worthy noting that the combination regimen exhibited the pronounced effect in this regard.

### 3.6. Effect of TAM, CAPE, and Their Combination on DNA Fragmentation in MCF-7 Cell Line

**Figure 6:**
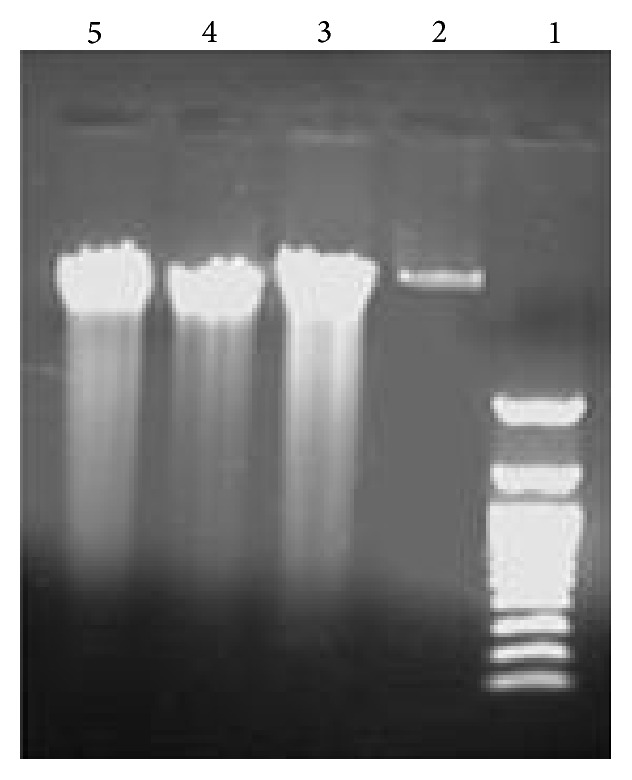
DNA gel electrophoresis in MCF-7 cells. DNA gel electrophoresis was performed after 48 h treatment with TAM (10 *μ*M), CAPE (4 *μ*M), and their combination. Lane (1): DNA ladder; lane (2): the control group; lane (3): TAM; lane (4): CAPE; lane (5): the combination therapy.

Gel electrophoresis revealed that treatment of MCF-7 cells with 10 *μ*M TAM, 4 *μ*M CAPE, and their combination for 48 h triggered degradation of DNA into oligonucleosomal fragments as detected by DNA laddering (Figure 6).

### 3.7. Gene Expression Profile

**Figure 7:**
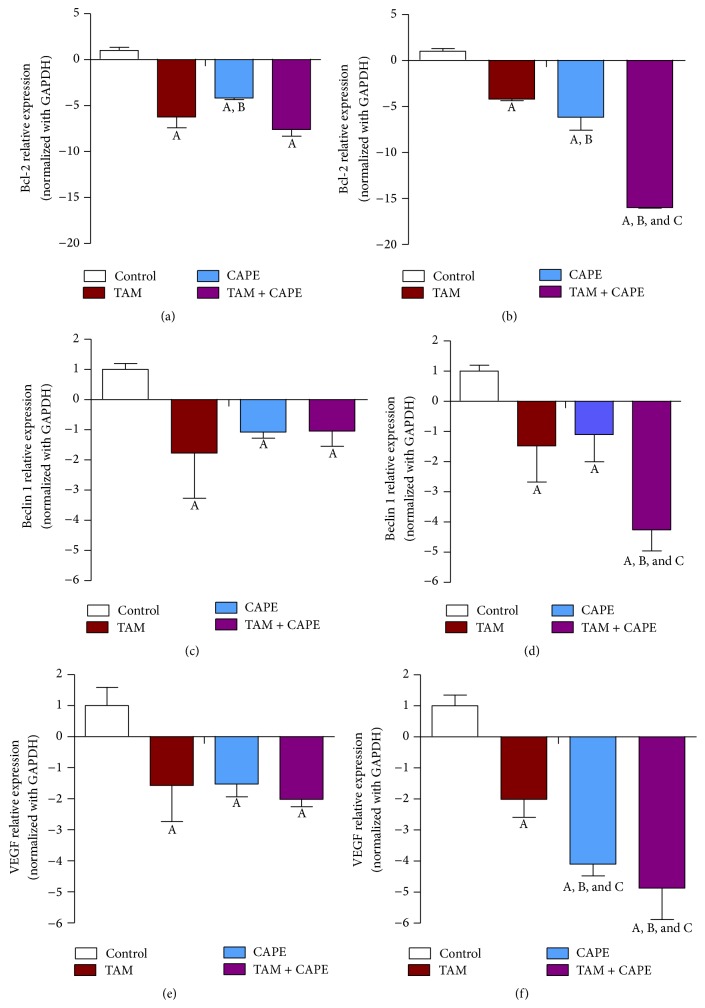
Expression levels of Bcl-2, Beclin 1, and VEGF in MCF-7 cells. Expression levels of Bcl-2, Beclin 1, and VEGF after 24 h (a, c, and e) and 48 h (b, d, and f) treatments with TAM (10 *μ*M), CAPE (4 *μ*M), and their combination. Results were expressed as relative expression of 3 independent experiments performed in triplets. Values of each bar are means ± SD. GAPDH was used as an internal control for calculating mRNA relative expression. A: significantly different from the respective concentration of control at *P* < 0.05. B: significantly different from the respective concentration of TAM at *P* < 0.05. C: significant difference of 48 h treatment from the respective concentration at 24 h at *P* < 0.05.

All treatments displayed a trend of downregulation in both Bcl-2 and Beclin 1 expression levels. As shown in Figure 7(a), 24 h treatment with either TAM or CAPE reduced Bcl-2 level almost 7- and 4-fold, respectively. Meanwhile, the combination afforded 8-fold decrease relative to the corresponding control values. On the other side, the 48 h (Figure 7(b)) treatment reduced its level 4-, 6-, and 15-fold for TAM, CAPE, and TAM + CAPE, respectively. Regarding Beclin 1 expression, treatment with TAM decreased its expression almost 2-fold after both periods. Meanwhile, the decrease was amounted to 1-fold in case of CAPE. Nevertheless, the combination regimen reduced its level 1-fold after 24 h and 4-fold after 48 h (Figures 7(c) and 7(d)). VEGF gene expression was also downregulated time dependently in all treated groups with the combination regimen showing the most potent effect (Figures 7(e) and 7(f)).

### 3.8. Effect of TAM, CAPE, and Their Combination on GSH Content in MCF-7 Cells

**Figure 8:**
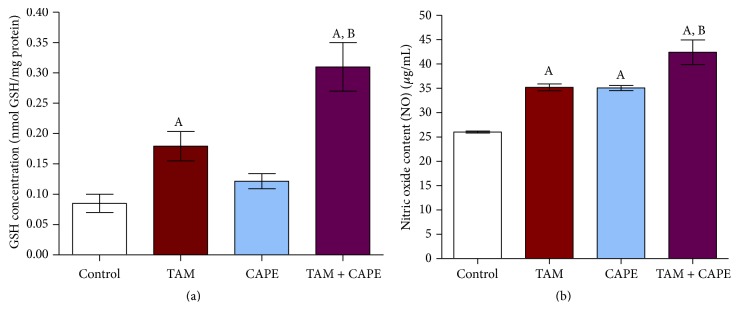
Glutathione (GSH) and nitric oxide (NO) levels in MCF7 cells following 48 h treatment. (a) Effect of treatment with 10 *μ*M TAM, 4 *μ*M CAPE, and their combination on glutathione (GSH) level in MCF-7 cells. The columns represent nmol GSH/mg protein. (b) Effect of treatment with 10 *μ*M TAM, 4 *μ*M CAPE, and their combination on nitric oxide (NO) level in MCF-7 cells. The columns represent *μ*g/mL nitrate. The values were represented as means ± SD of three separate experiments. A: significantly different from the control group at *P* < 0.05. B: significantly different from TAM group at *P* < 0.05.

As shown in Figure 8(a), 10 *μ*M TAM when used alone resulted in significant increase in cellular GSH content to approximately 2-fold as compared to the control. On the other hand, treatment with 4 *μ*M CAPE resulted in insignificant increase in GSH content relative to the control values. However, the combination of TAM and CAPE significantly increased GSH level almost 3-fold when compared to the control cells.

### 3.9. Effect of TAM, CAPE, and Their Combination on NO Production in MCF7 Cells

TAM and CAPE significantly increased NO content as compared to vehicle-treated cells (Figure 8(b)). However, the combination significantly increased the NO values compared to either single treatment group.

## 4. Discussion

Since cancer is still a major threat to health worldwide, there is global demand for more affordable and effective therapeutic alternatives. Perhaps, combining anticancer drugs with natural nutrients will be promising in the therapy of cancer patients.

According to this background, we aimed to explore the effect of TAM and CAPE, as a novel combination regimen in treating human breast cancer, and also to determine their supposed mechanisms of action.

The data of the present study revealed a concentration-dependent cytotoxic effect of TAM on MCF-7. This result supports the previously reported data elucidating the effectiveness and usefulness of TAM as a chemotherapeutic agent in human MCF-7 cells [24]. Similarly, the cytotoxic effect in MCF-7 treated with CAPE was evidenced herein. This finding concurs with the report of Wu et al. [14] demonstrating a diversity of oncolytic effects of CAPE in preclinical models of human breast cancer. Based on that, it is plausible that the combination of TAM and CAPE produced a synergistic cytotoxic effect in MCF-7 cells as indicated by CI.

Regarding the cellular uptake of TAM, the present data showed that the majority of the TAM was taken up by the cells after 2 h treatment and that no significant change in its uptake was achieved upon cotreatment with CAPE as compared with TAM treated group in all the studied time intervals. This can be explained on the basis of the fact that the lipophilicity of TAM is the controlling factor responsible for its cellular uptake [25]. TAM was taken up by the cells through simple diffusion until establishing equilibrium after 2 h, as suggested by the present data. De Santana et al. have previously reported that TAM citrate reached maximum cellular uptake after 4 h when tested in dogs [26]. This finding somewhat concurs with the current one.

Our data also revealed that the incubation of MCF-7 cells with TAM, CAPE, and their combination provoked the activation of caspase-9 and increased significantly caspase-3 activity. These results pointed to the contribution of the intrinsic pathway of apoptosis where cytosolic cytochrome c binds with procaspase-9 and Apaf-1, forming the apoptosome that triggers the cleavage of procaspase-9. Active caspase-9 then switches on downstream executioner caspases, including caspase-3 [27].

The roles of mitogen-activated protein kinases (MAPK), mitochondrial permeability transition, and ceramide generation have been formerly implicated in TAM-induced apoptosis [28]. Likewise, activation of Fas- and Bax-mediated apoptosis and associated DNA fragmentation was detected in MCF-7 human breast cancer cells following treatment with CAPE [29]. Thus, it is obvious that CAPE could potentiate both the antiproliferative and apoptotic effects of TAM.

It is worthy noting that despite the significant increase in caspase-9 activity, seen in the combination regimen after 24 h, there was a decrease in its activity after 48 h when compared to either TAM or CAPE alone. This result might be attributed to the increased GSH level observed in this group. Actually, increased GSH level decreased the availability of free radicals, leading to attenuation of mitochondrial-dependent apoptosis.

TAM- and CAPE-triggered apoptosis was also supported by DNA fragmentation in MCF-7 cells following treatments. This effect might be attributed to the caspases activation mentioned before. Virtually, DNA fragmentation is a consequence of the activation of a specific DNase (CAD, caspase activated DNase), found complexed with ICAD (inhibitor of CAD) in proliferating cells. When cells undergo apoptosis, caspases, in particular caspase-3, dissociate the CAD-ICAD complex, freeing CAD that cleaves chromosomal DNA [30].

Beclin 1 level plays a crucial role in controlling autophagy [31]. Another important factor involved in this process is the antiapoptotic Bcl-2, which is highly expressed in 40–80% of breast cancer patients [32]. Bcl-2 suppresses autophagy by directly targeting Beclin 1 [33].

In the present investigation, both Bcl-2 and Beclin 1 expression levels were downregulated in all treated groups. This finding pointed to their interaction. Interestingly, Beclin 1 was found to contain a well-recognized *α*-helical BH3 domain that allows it to dock into the hydrophobic groove of Bcl-2 [34]. Furthermore, decreased Beclin 1 expression level might be related to the plausible role of caspases in inactivating Beclin 1-induced autophagy. According to Wirawan et al. [34], caspases mediate cleavage of Beclin 1, abrogate its autophagic function, and generate a Beclin 1-C fragment that can enhance apoptosis by promoting the release of proapoptotic factors from the mitochondria. By this way, Beclin 1 switches from a proautophagic to a proapoptotic protein. In this context, Qadir et al. [35] have demonstrated that when autophagy is compromised in TAM treated MCF-7 cells, activation of the mitochondrial apoptotic pathway takes place and increased apoptosis is achieved, at least in part, through caspase-9.

Herein, no change in the protein level of LC3-II was detected in all treated groups. These results were in contrast with those of Hwang et al. [36] who found increased levels of LC3-II in MCF-7 cells exposed to TAM. Similarly, CAPE triggered activation of the autophagic response in C6 glioma cells by inducing an increase in LC3 [37].

The discrepancy between our result and other researchers may be due to time of detection of autophagosomes and interplay between autophagy and other players as oxidative stress, metabolites, and apoptosis.

The major problem was to quantify precisely autophagy levels in cancer cells. Indeed, LC3-II levels have been described as an accurate marker of the number of autophagosomes in cells. However, an accumulation of these intracellular vesicles can be also linked to an increase of autophagy induction or an inhibition of autophagosome degradation by the lysosomes. To our knowledge, no autophagy marker is currently available in order to discriminate between an increase or a decrease of overall autophagy flux* in vivo* [38].

The present study also wanted to clarify whether, or not, the tumor inhibitory effect of TAM + CAPE is mediated via angiogenesis inhibition in breast cancer. Previously, low serum VEGF levels were reported in breast carcinoma patients after TAM therapy [39]. However, the marked decrease in VEGF levels when combined with CAPE could be related to the additional angiostatic activity exerted by CAPE. According to El-Refaei and El-Naa [40], CAPE controls tumor growth by elevating the angiostatic factors and inhibiting the angiogenic ones. Moreover, CAPE was able to inhibit NF-*κ*B in human breast cancer MCF-7 cells [29]. Blockade of NF-*κ*B signaling has been suggested to inhibit angiogenesis and tumorigenicity in different types of cancer cells by suppressing the expression of VEGF [41].

Unexpectedly, the current study demonstrated that both TAM and CAPE alone increased GSH content, whereas their combination enhanced this effect compared to either treatment alone. These data suggested that increased cellular GSH content might have a role in TAM and CAPE-mediated effect. In line, Loo et al. [42] and Moreira et al. [43] have demonstrated that TAM exhibited an antioxidant effect and was able to induce the NO synthesis* in vitro*. Likewise, Singhal et al. [44] have revealed that low concentrations of polyphenolics can enhance the activity of *γ*-glutamyl cysteinyl synthetase and other GSH-linked detoxifying enzymes, preserving thus GSH level.

With respect to the effect of TAM and CAPE on NO production, we found an increase in NO level. Previously, TAM treatment has prevented the transformation of C3H10T1/2 murine fibroblast cell line and has augmented NO production via the induction of inducible nitric oxide synthase (iNOS) at concentrations blocking the cell transformation [42]. NO was found to exert a cytotoxic effect on tumor cells via inhibition of cellular proliferation [45]. Moreover, Ignarro et al. [46] have confirmed the inhibitory effect of arginine-NO pathway in vascular smooth muscle proliferation and have attributed their finding to the capacity of NO to inhibit ornithine decarboxylase. Furthermore, it has been reported that NO possesses a cytotoxic effect caused by its interaction with the Fe-containing rate-limiting enzyme of DNA synthesis, ribonucleotide reductase [47], and [Fe-S] cluster enzymes, as mitochondrial aconitase [48, 49]. Moreover, Watts et al. [50] have reported that the cytotoxic and the antiproliferative effects of NO were attributed to its ability to induce iron and GSH efflux from tumor cells via the GSH transporter multidrug resistance-associated protein 1 (MRP1).

In conclusion, this study demonstrates that CAPE enhanced TAM cytotoxicity via multitarget approach, including weakening of autophagy, strengthening of both apoptotic and angiostatic potentials, and finally augmentation of both cellular GSH and NO levels. In addition, the ability of CAPE to lower the effective dose of TAM provides a rationale for further experimental and clinical investigations of this combination.

## Figures and Tables

**Table 1:** Primer sequences for quantitative real time PCR.

Gene	Sequences
Bcl-2	Forward 5′-TCT GAC GGC AAC TTC AAC TG-3′
	Reverse 5′-TGG GTG TCC CAA AGT AGG AG-3′

Beclin 1	Forward 5′-ATC CTG GAC CGT GTC ACC ATC CAG G-3′
	Reverse 5′-GTT GAG CTG AGT GTC CAG CTG G-3′

VEGF	Forward 5′-TCC TCA CAC CAT TGA AAC CA-3′
	Reverse 5′-GAT CCT GCC CTG TCT CTC TG-3′

GAPDH	Forward 5′-CAA GGT CAT CCA TGA CAA CTT TG-3′
	Reverse 5′-GTC CAC CAC CCT GTT GCT GTA G-3′
